# P-175. Clinical Profile and Management Outcomes of Isolated Myocysticercosis: A Prospective Observational Cohort Study from a Tertiary Care Center in India

**DOI:** 10.1093/ofid/ofaf695.399

**Published:** 2026-01-11

**Authors:** Baidhnath Kumar Gupta, Sayan Maharatna, Satish Swain, Charu Rajoria, Manish Soneja, Insha Insha, Stanzin Spalkit

**Affiliations:** All India Institute of Medical Sciences, New Delhi, Delhi, Delhi, India; All India Institute of Medical Sciences, New Delhi, Delhi, Delhi, India; All India Institute of Medical Sciences, New Delhi, Delhi, Delhi, India; All India Institute of Medical Sciences, New Delhi, Delhi, Delhi, India; All India Institute of Medical Sciences, New Delhi, Delhi, India; All India Institute of Medical Sciences, New Delhi, Delhi, Delhi, India; All India Institute of Medical Sciences, New Delhi, Delhi, Delhi, India

## Abstract

**Background:**

Cysticercosis, caused by *Taenia solium* larvae, remains endemic in Asia and Latin America. Isolated myocysticercosis—confined to skeletal muscle—is uncommon and often misdiagnosed due to nonspecific symptoms. In India, data on isolated muscular involvement is limited, usually restricted to case reports.

**Figure:**
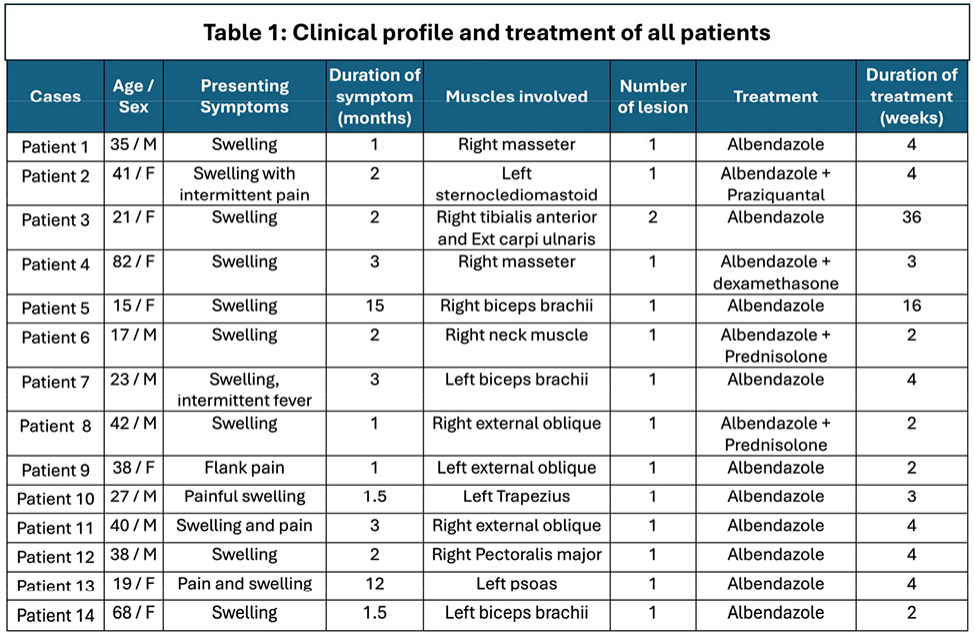

**Figure:**
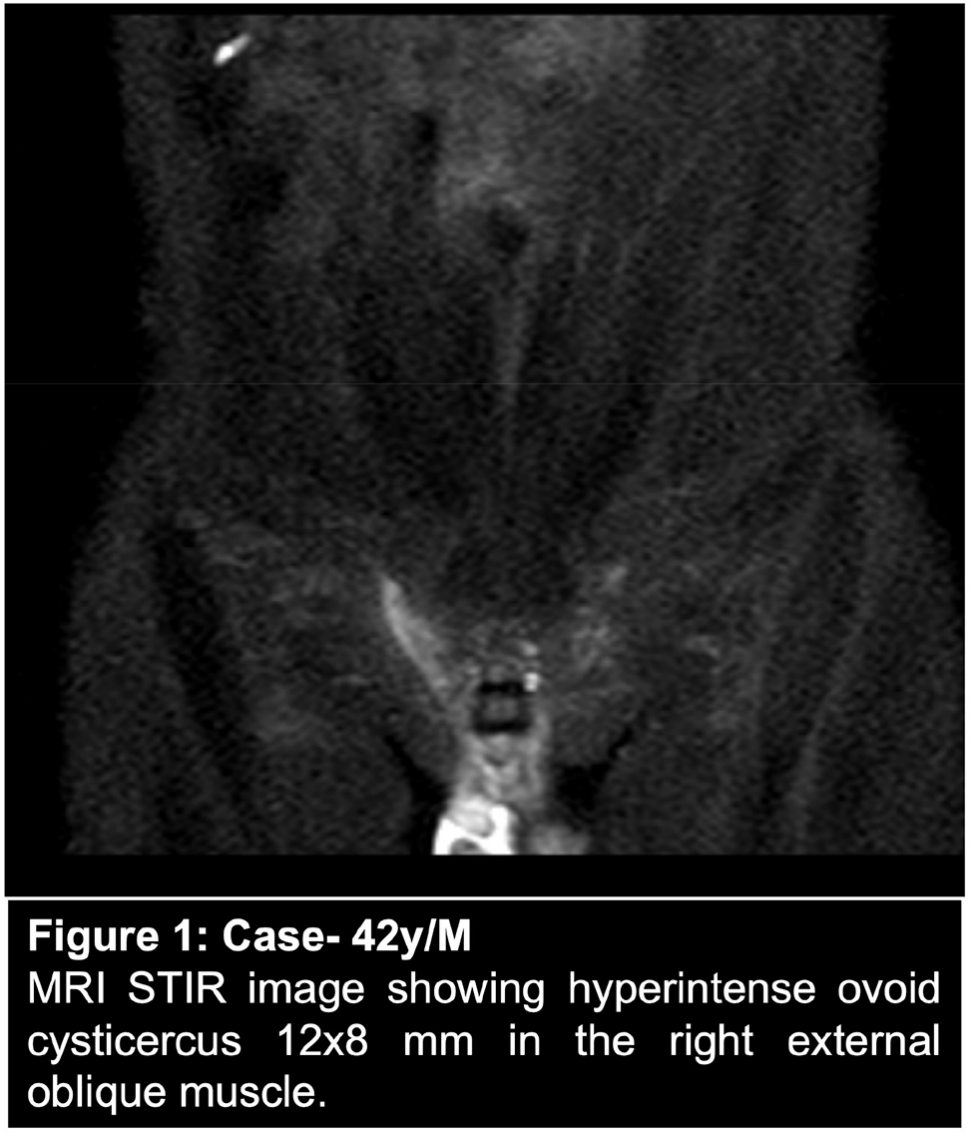

**Methods:**

This prospective observational study was conducted at a North Indian tertiary center (Jan 2022–July 2024). Patients with muscular swellings diagnosed as myocysticercosis via clinical, radiological (USG/MRI), and/or histopathological criteria were enrolled after informed consent.

Inclusion: Isolated muscular involvement without CNS lesions.

Exclusion: Neurocysticercosis or inadequate follow-up (< 1 month).

Patients were monitored for clinical/radiological resolution, complications, and relapse.

**Figure:**
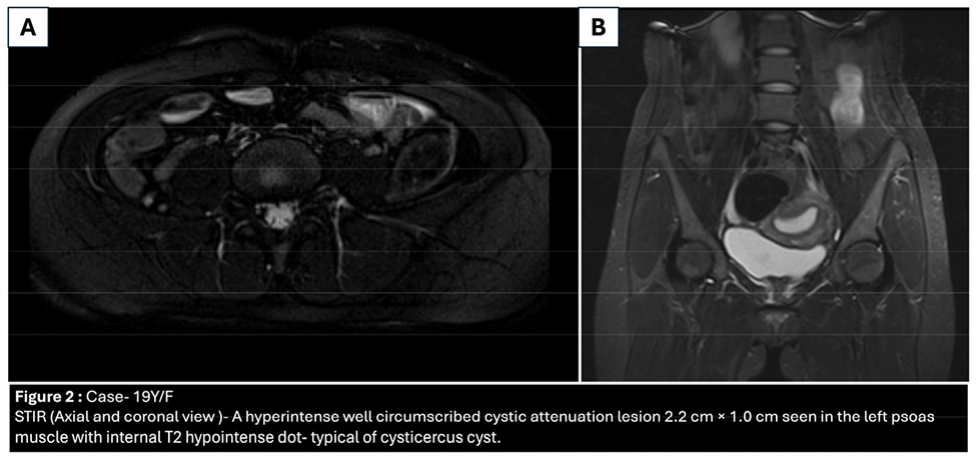

**Results:**

Fourteen patients (7 males, 7 females), mean age 36.1 years, were included. Most (86%) presented with swelling; others had pain (3) or fever (1). Thirteen had solitary lesions; commonly involved muscles were biceps (3), masseter (2), external oblique (2). Ten patients (71%) were vegetarians. Nine (64%) had no comorbidities; others had diabetes (3), migraine (1), or thyroid swelling (1).

All underwent fundoscopy and brain/orbit imaging to exclude neuro/ocular cysticercosis. Albendazole was used in all; three received combination therapy (with praziquantel or steroids). Median treatment duration was 3.5 weeks. Resolution with calcification occurred in 9 patients (64%). None required surgery or showed relapse over ≥6 months of follow-up.

**Conclusion:**

Isolated myocysticercosis presents as localized muscular swelling, affects diverse muscles, and occurs regardless of diet. Albendazole-based therapy is effective. This study highlights the clinical spectrum, muscle involvement, and non-surgical resolution, underscoring the need for standardized management protocols and multicenter data.

**Disclosures:**

All Authors: No reported disclosures

